# Compensating elastic faults in a torque-assisted knee exoskeleton: functional evaluation and user perception study

**DOI:** 10.1186/s12984-024-01531-6

**Published:** 2024-12-28

**Authors:** Rodrigo J. Velasco-Guillen, Adna Bliek, Josep M. Font-Llagunes, Bram Vanderborght, Philipp Beckerle

**Affiliations:** 1 Chair of Autonomous Systems and Mechatronics, Friedrich-Alexander University Erlangen-Nürnberg, Erlangen, Germany; 2 Biomechanical Engineering Lab, Department of Mechanical Engineering and Research Centre for Biomedical Engineering, Universitat Politècnica de Catalunya, Barcelona, Spain; 3 Health Technologies and Innovation, Institut de Recerca Sant Joan de Déu, Esplugues de Llobregat, Spain; 4 Robotics and MultiBody Mechanics Research Group, Vrije Universiteit Brussel, Brussels, Belgium; 5 Interuniversity Microelectronics Centre (imec), Leuven, Belgium; 6 Department Artificial Intelligence in Biomedical Engineering (AIBE), Friedrich-Alexander University Erlangen-Nürnberg, Erlangen, Germany

**Keywords:** Adaptive control, Elastic actuation, Fault tolerance, Human-robot interaction, Impedance control, Wearable robotics

## Abstract

Wearable robots are often powered by elastic actuators, which can mimic the intrinsic compliance observed in human joints, contributing to safe and seamless interaction. However, due to their increased complexity, when compared to direct drives, elastic actuators are susceptible to faults, which pose significant challenges, potentially compromising user experience and safety during interaction. In this article, we developed a fault-tolerant control strategy for torque assistance in a knee exoskeleton and investigated user experience during a walking task while emulating faults. We implemented and evaluated the torque control scheme, based on impedance control, for a mechanically adjustable compliance actuator with nonlinear torque-deflection characteristics. Conducted functional evaluation experiments showed that the control strategy is capable of providing support during gait based on a torque profile. A user study was conducted to evaluate the impact of fault severity and compensation on the perception of support, stiffness, comfort, and trust while walking with the exoskeleton. Results from the user study revealed significant differences in participants’ responses when comparing support and stiffness levels without fault compensation. In contrast, no significant differences were found when faults were compensated, indicating that fault tolerance can be achieved in practice. Meanwhile, comfort and trust measurements do not seem to depend directly on torque support levels, pointing to other influencing factors that could be considered in future research.

## Introduction

The field of wearable robotics has witnessed significant advancements in recent years. In particular, powered exoskeletons have garnered attention for their potential to augment or restore human joint function [1]. Elastically actuated systems present a viable solution by mimicking the intrinsic compliance observed in natural human joints, contributing to a seamless interaction between the wearer and the exoskeleton. The Series Elastic Actuator (SEA), in which input and output are coupled in series by an elastic element, is a popular topology choice for wearable robots due to the added safety through intrinsic output backdrivability, its energy efficiency capabilities, and its intrinsically low output impedance [2–5]. In SEAs, the relationship between torque and deflection effectively turns the torque control problem into a position control problem. In other words, a controller may be used to adjust the input position to attain a desired output torque, which can improve force accuracy since controlling position accurately in geared motors is generally easier than force [6]. This method of indirect force control in SEAs is common in literature [7–9], but it generally requires accurate knowledge of the series elasticity properties. The Parallel Elastic Actuator (PEA) topology, in which an elastic element is connected in parallel to the input, is a less popular choice, but it has shown advantages such as torque-control performance and energy efficiency in task specific scenarios [10–12]. Elastic actuators can also be designed with fixed or variable stiffness characteristics. Variable Stiffness Actuators (VSAs) are devices capable of adjusting their stiffness through mechanisms that alter elastic properties [13] and are also often used in wearable robots due to their adaptable mechanical properties [14, 15].

However, the mechanical complexity of elastic actuators increases compared to direct drives, e.g., due to the inclusion of elastic and kinematic elements. Through that, the chance of fault occurrence, in general, increases [16]. The reliability of robotic systems strongly depends on their capability to maintain nominal operation in the presence of unexpected conditions [17]. Internal faults, such as abrupt changes of internal properties, can lead the system to undesired or unsafe configurations. From a technical perspective, the ideal case would be to design a system which is inherently fault-free. However, this is practically unachievable, and fault tolerance is a concept which aims at incorporating redundancy concepts and/or control reconfiguration to avoid these internal faults from causing service failure [17, 18]. The study in [16] highlighted the practical relevance of faults in elastic actuators. While stiffness degradation in elastic elements might occur due to cyclic loading or excessive forces, faults in kinematic components such as linkages and cables, may be more frequent in elastically actuated systems [16]. This is particularly critical for VSAs which introduce kinematic elements in their stiffness adjusting mechanisms. Elastic faults are then defined as internal changes that may alter the elastic behavior of the actuator and thus its effective stiffness and output torque. Before they could severely impact the system functionality, a fault-tolerant control strategy can be implemented to detect and compensate for such faults [18]. In literature, adaptive control methods are an alternative to achieve fault-tolerance in the face of uncertain parameters in elastic actuators by applying robust methods such as disturbance observers [19, 20] or parameter estimation [7, 21].

The main research question tackled in this paper is how users experience and react to the impact of elastic faults on physical human-robot interaction (pHRI). As such, we investigate the torque support performance and user perception during walking with an elastically powered knee exoskeleton, while emulating elastic faults. This serves as a scenario of close physical interaction and allows to evaluate whether fault tolerance can be achieved in practice through fault compensation and to gain insight in the influencing human factors through psychometric experiments. One critical aspect of exoskeleton control is providing appropriate support to the user. Support refers to the assistance or resistance provided by the exoskeleton’s actuators to help the user perform tasks more effectively or safely, which can significantly impact the user’s experience while using the exoskeleton. Research shows that exoskeleton support can improve user performance and decrease task difficulty and fatigue [22]. However, faulty conditions might degrade the provided support and affect the user experience in the interaction. Investigating user experience with exoskeletons allows to understand how users might experience and interact with the forces applied by the exoskeleton during movement tasks. Fault tolerance in exoskeletons is important to ensure that users experience consistent support and are not disturbed by potential faults. Properly compensating for faults should result in users perceiving no changes in the exoskeleton’s performance, maintaining seamless assistance during use. At the same time, users may not immediately notice minor variations in forces or torques, so understanding what degree of fault severity is noticeable is also important. This knowledge helps in designing exoskeletons that can maintain high performance and reliability even in the presence of faults, thereby enhancing user safety and confidence. A compliant control method, such as impedance control, is a suitable solution for providing the required support while allowing for adaptability in the pHRI behavior [23, 24].

**Fig. 1:**
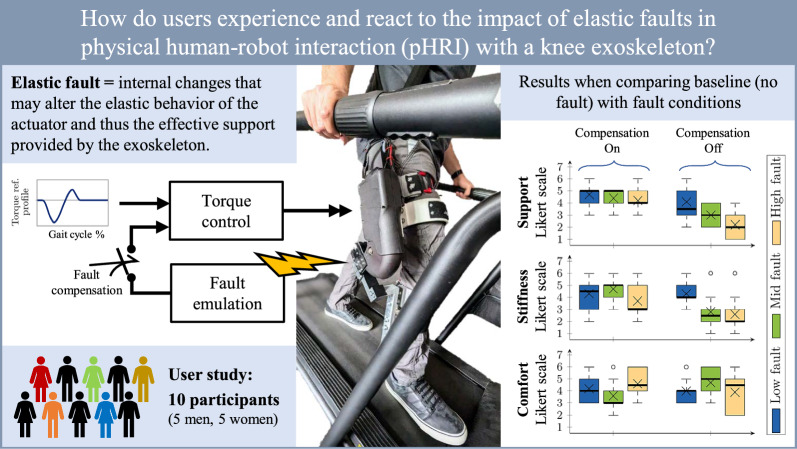
Main contributions of the paper. The primary research question, methods, and results are summarized. Results from the user study conducted showed that, when faults are compensated, participants did not distinguish faulty conditions in terms of support and stiffness, suggesting that fault tolerance is achieved. Meanwhile, for uncompensated faults, participants clearly distinguished mid and high faulty conditions

This article extends previous research on fault-tolerant pHRI in elastic actuators [25–27], where a trajectory tracking control strategy based on an established passivity-based impedance controller from [28] was implemented. The control strategy was capable of adapting a desired interaction impedance behavior by detecting and compensating faults in elastic elements using online stiffness estimation. Specifically, we contribute by adapting the strategy to providing torque support, and investigate the impact of compensated and uncompensated faults on user perception when interacting with a SEA-powered knee exoskeleton. We explore the theoretical foundations and experimental validation of the proposed control method for providing a gait torque support profile to users through the knee exoskeleton, and present a user study to evaluate how users experience changes in gait support due to faults. The study investigated the impact on the perceived support level, stiffness, comfort, and trust of several users while physically interacting with the knee exoskeleton in locomotion under different fault conditions. The main contributions of the paper are summarized in Fig. 1.

The rest of the article is organized as follows. Section "Fault-tolerant torque control" details the torque control strategy. Section "Materials and methods" describes the hardware setup, experimental evaluation, and user study design. Section "User study results" presents the results of the user study. Findings are discussed in Sect. "Discussion". Finally, conclusions are presented in Sect. "Conclusions".

## Fault-tolerant torque control

**Fig. 2:**
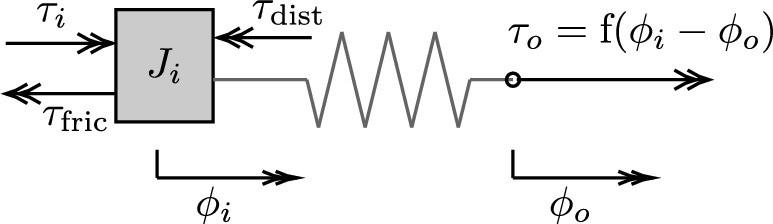
Diagram of a general SEA with the output torque $\tau _o$ as a nonlinear torque function of its deflection

Consider that, in a general case, a rotary series elastic actuator (SEA), shown in Fig. 2, has an input position $\varphi _i$ and output position $\varphi _o$, and its output torque $\tau _o$ completely defined by a nonlinear elastic torque function which depends on its deflection $\alpha =\varphi _i-\varphi _o$. The actuator dynamics can then be defined by

1 $$\begin{aligned} J_i\ddot{\varphi }_i&=\tau _i-\tau _o-\tau _\text {fric}-\tau _\text {dist}\,, \\ \text {with } \tau _{o}&= \text {f}(\alpha )\,,\nonumber \end{aligned}$$

where $J_i$ is the input-side inertia, $\tau _i$ is the input torque provided by the motor, $\tau _\text {fric}$ is the friction torque, and $\tau _\text {dist}$ represents torque caused by disturbances.

Given the relationship between torque and deflection in SEAs, torque control can be implemented indirectly by controlling the motor position. A desired torque reference $\tau _{o,d}$ can be obtained by computing a reference input position $\varphi _{i,d}$ such that

2 $$\begin{aligned} \tau _{o,d}=\text {f}(\alpha _d), \quad \varphi _{i,d}=\alpha _d+\varphi _{o}\,. \end{aligned}$$

**Fig. 3:**
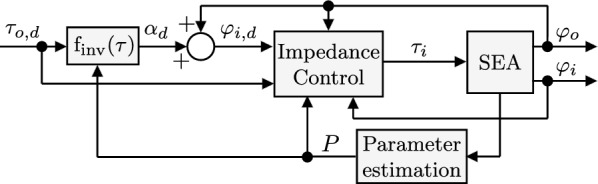
Torque assistive control strategy for the nonlinear series elastic actuator that drives the knee exoskeleton. A desired torque $\tau _{o,d}$ is tracked by transforming the torque reference into a desired deflection $\alpha _d$ and to a desired actuator trajectory $\varphi _{i,d}$. A passivity-based impedance controller is then used to track the reference and attain the desired torque. A parameter estimation algorithm is used to observe the spring pretension parameter *P* which is modulated to emulate faults

The block diagram of the torque control strategy is shown in Fig. 3. The desired torque $\tau _{o,d}$ is transformed into a reference deflection $\alpha _d$ by utilizing the inverse torque function $\text{f}_\text {inv}(\tau _{o,d})=\alpha _d$. However, since it might not be possible to describe the inverse of nonlinear function analytically, one can resort to finding the root of the auxiliary function $\text {h}(\alpha ) = \text {f}(\alpha )-\tau _{o,d}$ at each time step using the Newton–Raphson method iteratively at discrete instances $i=0,1,2,\dots$:

3 $$\begin{aligned} \alpha _{i+1} = \alpha _i + \frac{\text {h}(\alpha _i)}{\text {h}'(\alpha _i)}\,, \end{aligned}$$

where $\text {h}'(\alpha )=\frac{\partial \text {f}(\alpha )}{\partial \alpha }$. The process is repeated until the root is found (when the absolute value of $\text {h}(\alpha _i)$ is below a threshold) or a maximum number of iterations is reached. The value of the last iteration then corresponds to $\alpha _d$ and the reference input position $\varphi _{i,d}=\alpha _d+\varphi _o$ is tracked by an impedance controller.

### Impedance controller

Now consider the following control law, adapted from [28], of a passivity-based impedance controller for trajectory tracking with inertia shaping and friction compensation:

4 $$\begin{aligned} \tau _i = \frac{J_i}{J_{i,d}}u+\left( 1-\frac{J_i}{J_{i,d}}\right) \tau _o+\tau _\text {fric}\,, \end{aligned}$$

where

5 $$\begin{aligned} u = J_{i,d}\ddot{\varphi }_{i,d}+ \tau _{o,d}+k_c(\varphi _{i,d}-\varphi _i)+d_c(\dot{\varphi }_{i,d}-\dot{\varphi }_i)\,. \end{aligned}$$

Note that the first and second terms of the control variable in eq. (5) perform feedforward inertial and elastic torque compensation, respectively; while the third and fourth terms function as a proportional-derivative controller with the proportional value $k_c$ acting as a virtual spring with equilibrium position at $\varphi _{i,d}$, and the derivative value $d_c$ acting as a virtual damper with equilibrium position at $\dot{\varphi }_{i,d}$.

### Closed-loop dynamics

Applying the control law in eq. (4) to the actuator dynamics in eq. (1) attains the following closed-loop dynamics:

6 $$\begin{aligned} J_{i,d}\ddot{\tilde{\varphi }}_i+d_c \dot{\tilde{\varphi }}_i+k_c \tilde{\varphi }_i&=-\tilde{\tau }_o + \frac{J_{i,d}}{J_i}\tau _\text {dist}\,, \end{aligned}$$

where $\tilde{\varphi }_i=\varphi _{i,d}-\varphi _i$ and $\tilde{\tau }_o = \tau _{o,d}-\tau _o$. In order to relate the equation to only $\tilde{\varphi }_i$ or $\tilde{\tau _o}$, we should consider the linearized case: $\tilde{\tau }_o \approx k_s \tilde{\varphi }_i$, where $k_s$ corresponds to the instantaneous stiffness constant:

7 $$\begin{aligned} k_s=\frac{\partial \text {f}(\alpha )}{\partial \alpha }\,. \end{aligned}$$

The closed-loop dynamics can now be expressed as

8 $$\begin{aligned} J_{i,d}\ddot{\tilde{\varphi }}_i+d_c \dot{\tilde{\varphi }}_i+(k_c+k_s)\tilde{\varphi }_i= \frac{J_{i,d}}{J_i}\tau _\text {dist}\,, \end{aligned}$$

or

9 $$\begin{aligned} \frac{J_{i,d}}{k_s}\ddot{\tilde{\tau }}_o+\frac{d_c}{k_s}\dot{\tilde{\tau }}_o+\left( \frac{k_c}{k_s}+1\right) \tilde{\tau }_o=\frac{J_{i,d}}{J_i}\tau _\text {dist}\,. \end{aligned}$$

Notice that for the non-disturbed case, i.e., $\tau _\text {dist}=0$, the closed-loop dynamics for both position and torque are described by a second order homogeneous linear differential equation with natural frequency $\omega _0$ and damping ratio $\delta$:

10 $$\begin{aligned} \omega _0 = \sqrt{\frac{k_c+k_s}{J_{i,d}}}\,,\quad \delta = \frac{d_c}{2\omega _0 J_{i,d}}\,. \end{aligned}$$

The proposed approach is based on the trajectory tracking control strategy from our previous research [26], which utilizes the same impedance control algorithm but the input reference is computed based on the reference output trajectory. In this work, the strategy is adapted for torque control, which is a very common approach for lower limb assistive robotic devices [29], by computing an input reference based on the output feedback and a reference torque that is ideally tracked regardless of the selected virtual impedance components. The control parameters $J_{i,d}$, $d_c$, and $k_c$ can be, in principle, tuned to obtain a critically damped response.

### Disturbance rejection

Analyzing the disturbance rejection, the transfer function of the linearized dynamics in eq. (9) is the following:

11 $$\begin{aligned} D(s)=\frac{\tilde{\tau }_o(s)}{\tau _\text {dist}(s)}=\frac{J_{i,d}}{J_i}\left( \frac{k_s}{J_{i,d}s^2+d_cs+k_c+k_s}\right) \,. \end{aligned}$$

Note that for a low operating frequency

12 $$\begin{aligned} \lim _{s\rightarrow 0} D(s)= \frac{J_{i,d}}{J_i}\left( \frac{k_s}{k_c+k_s}\right) \,. \end{aligned}$$

From this we can notice that the disturbance rejection is lower for a stiffer system, but it also improves by selecting a high virtual stiffness $k_c$.

The control law in eq. (4) allows to virtually shape a desired inertia $J_{i,d}$. Inertia shaping is effectively engaged in instances where $J_{i,d}\ne J_i$. Typically, a reduction in inertia, i.e, $J_{i,d}\le J_i$ is advantageous. Diminishing inertia results in an elevated multiplier for the control input, thereby enhancing the system’s responsiveness. Our prior research demonstrated that opting for a lower desired inertia yields favorable outcomes in haptic perception [30]. It is important to observe that this reduction in inertia concurrently diminishes disturbance rejection by a commensurate factor, thereby augmenting the system’s resilience to unmodeled or disruptive behaviors.

### Fault compensation

The adaptability of the proposed model-based approach allows to accommodate faults as uncertain parameters. As mentioned in Sect. "Introduction", elastic faults are conceptualized as changes in the anticipated behavior of the elastic torque function $\text {f}(\alpha )$ caused by changes in the internal parameters which depend on the actuator design (e.g., spring pretension). Accurate fault detection, in this case, requires to identify and observe relevant uncertain parameters.

In this work, an online parameter estimation method, adapted from [31], is applied, where an unscented Kalman filter (UKF) is used to estimate the spring pretension *P* in a SEA with a nonlinear torque-deflection relationship, described in Sect. "Knee exoskeleton". Changes in pretension emulate stiffness degradation due to the deformation of the spring, and/or faults in the kinematic elements that comprise the tensing mechanism that adds pretension to the linear spring, e.g., the deformation of the connecting cable. In practice, the method can be applied to estimate any internal parameter that alters the elastic torque function and is subject to changes due to faults. The online estimation method is applied at discrete time instances $n=0,1,2\dots$ and requires that the output torque at a particular time instance $\tau _{o,n}$ is known or measured. The estimation is then performed considering that the elastic torque function depends on both the deflection and the internal parameter, in this case the pretension: $\tau _{o,n}=\text {f}(\alpha _n,P_n)$.

Our filter was designed with the pretension as the state value $x_n=P_n$, the deflection as input $u_n=\alpha _n$, and the elastic torque as the measured output $y_n=\tau _{o,n}$. The UKF algorithm considering additive (zero mean) noise from [32] was then used to find the estimated state $\hat{x}_n=\hat{P}_n$, considering the state and measurement functions $\text {F}(x_n,u_n)=P_n$ and $\text {H}(x_n,u_n)=\text {f}(\alpha _n,P_n)$, respectively.

During experimentation with active fault detection, to avoid delays in the convergence of the estimated pretension to the reference value, the UKF algorithm is restarted after each pretension change, with the expected pretension as the initial value. The UKF algorithm was internally parameterized based on the recommendations from [32]. The measurement noise covariance was set to $R=0.7$, while the process noise covariance was tuned for smooth and slow dynamics to $Q= {1e-12}$.

A fault is *compensated* when the parameter estimation algorithm is active and the precise values of the uncertain parameter is known and considered in control calculations, while the fault is *uncompensated* when the parameter is set at a constant baseline value (no fault detection), which makes the determination of the reference input position $\varphi _{i,d}$ and the control law prone to inaccuracy, potentially leading to a deviation from the desired system behavior.

## Materials and methods

This section details the material and methods utilized to evaluate the control strategy presented in Sect. "Fault-tolerant torque control" to achieve fault tolerance in a knee exoskeleton as a scenario of tight pHRI interaction. The knee exoskeleton is presented, followed by an experimental functional evaluation with a single user, and finally a user study design to evaluate fault tolerance and user interaction with multiple participants.

### Knee exoskeleton

**Fig. 4:**
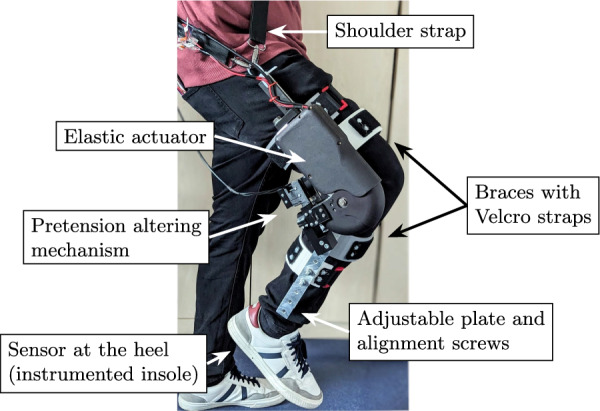
Knee exoskeleton experimental setup. The elastic actuator, which drives the exoskeleton, is equipped with a pretension altering mechanism to emulate faults by changing the elastic behavior of the actuator. The exoskeleton is attached to the user using braces with Velcro straps and adjustable plates with alignment screws to customize the brace positions in the proximal/distal and medial/lateral directions. A shoulder strap is included to support the weight of the device. An instrumented insole, embedded with a force-sensing resistor, is used to detect heel strike

Figure 4 shows the design of the knee exoskeleton for experimentation on a treadmill. Powered by the SMARCOS actuator [33], a mechanically adjustable compliance and controllable equilibrium position actuator (MACCEPA), the exoskeleton attaches to the leg segments using braces 3D-printed with technical flexible filament (Flexfill TPU) and secured with Velcro straps. Adjustable plates and alignment screws allow to customize the brace positions in the proximal/distal and medial/lateral directions to ensure that the axis of rotation of the actuator aligns with that of the knee of the user. A shoulder strap supports the weight of the device (4.05 kg) during walking. Finally, the elastic properties of the actuator can be modulated by altering the pretension of a linear spring using a servomotor, effectively emulating elastic faults.

**Fig. 5:**
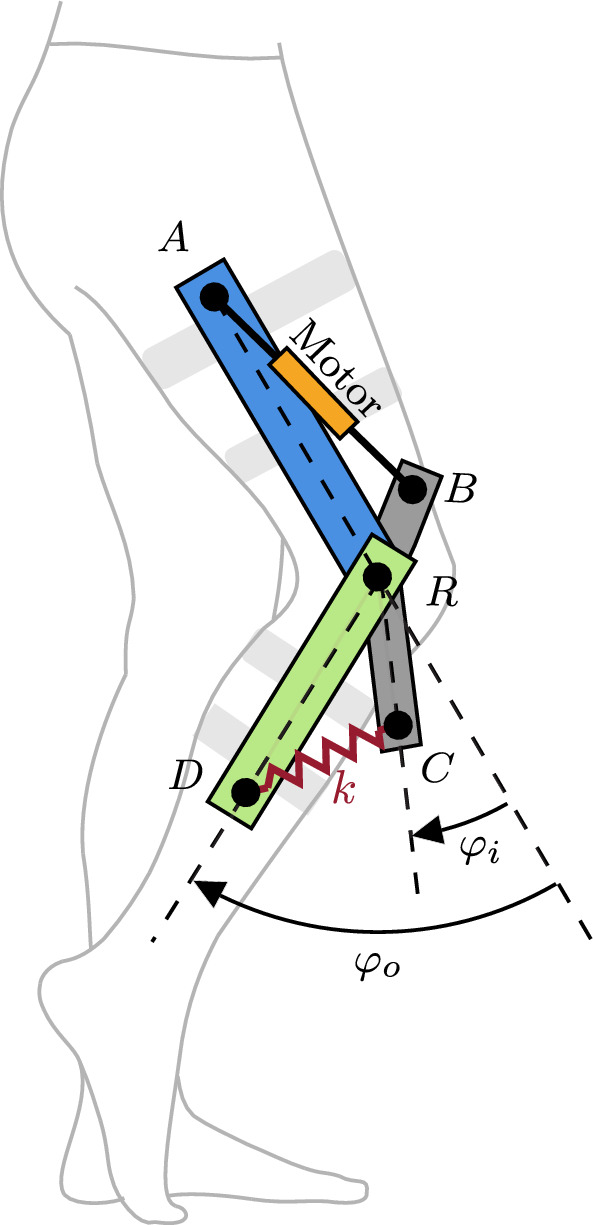
Kinematic model of the MACCEPA-based actuator

The actuator configuration, shown in Fig. 5, consists of an input link between points *A* and *R*, an output link between points *C* and *R*, and a lever arm between points *B*, *R*, and *D*, all rotating around point *R*. The input link and the lever arm are rigidly coupled through a motorized screw drive between points *A* and *B*. Moreover, a linear spring with stiffness *k* is connected between points *C* and *D*. The positions of the lever arm $\varphi _i$ and output link $\varphi _o$ are measured with magnetic encoders, their speeds are computed with filtered numerical derivation, and the elastic torque $\tau _o$ is indirectly computed from force measurement at the base of the motor.

The output torque $\tau _o$ created by the spring due to the deformation $\alpha = \varphi _i-\varphi _o$ can be computed based on the geometric properties of the actuator [34]:

13 $$\begin{aligned} \tau _o = \text {f}(\alpha ) = kL_CL_D\sin {\alpha }\left( 1+\frac{P-\left| L_D-L_C\right| }{L_{CD}}\right) ,\\ \text {with } L_{CD}=\left( L_C^2+L_D^2-2L_CL_D\cos {\alpha }\right) ^{1/2}\,,\nonumber \end{aligned}$$

where $L_C$ is the length between points *C* and *R*, $L_D$ is the length between points *D* and *R*, and *P* corresponds to the pretension length of the spring. The solution of eq. (13) for $\alpha$ is not analytically possible. Therefore, the Newton–Raphson iterative method, described in Sect. "Fault-tolerant torque control", is used to find a numerical solution.

The behavior of the MACCEPA is that of a SEA with a nonlinear torque function. At a given deflection, the apparent stiffness of the actuator can be computed based on eq. (7) as

14 $$\begin{aligned} k_{s} = kL_CL_D\cos {\alpha }\left( 1+\frac{P-\left| L_D-L_C\right| }{L_{CD}}\right) -kL^2_CL^2_D\sin ^2{\alpha }\left( \frac{P-\left| L_D-L_C\right| }{L_{CD}^3}\right) . \end{aligned}$$

Modifying the pretension *P* alters the behavior of $\text {f}(\alpha )$ and $k_s$ and thus emulates elastic faults as they might be caused by plastic deformations in the spring or cables in the coupling mechanism.

### Functional evaluation experiments

We tested the performance of the exoskeleton while walking on a treadmill at a fixed speed in two different conditions: In *zero-torque mode*, the provided support is zero, i.e., the user can walk freely, ideally without resistance; and in *support mode*, a torque profile is provided in synchronism with the gait cycle. Experiments were carried out with one experienced user (male, 177 cm, 90 kg) walking on a treadmill at 0.58m/s. The synchronization of gait cycle was triggered by a force-sensing resistor (FSR) embedded in an instrumented insole, which detects heel strike as the beginning of the gait cycle. For simplicity, and to avoid introducing noisy signal derivatives to the control law, we assume that the position reference $\varphi _{i,d}$ is static, i.e., $\dot{\varphi }_{i,d}=0$ and $\ddot{\varphi }_{i,d}=0$ .

The friction is modeled combining dry (Coulomb) and viscous components [35]:

15 $$\begin{aligned} \tau _\text {fric} = c_{c}{{\,\textrm{sign}\,}}{\dot{\varphi }} + c_v\dot{\varphi }\,, \end{aligned}$$

where $c_c$ and $c_v$ are the Coulomb and viscous friction coefficients, respectively. The parameters of the actuator, extracted from [26] and [33], and the control parameters used in the experimentation are detailed in Table 1.

**Table 1:** Parameters of the MACCEPA-based actuator

Description		Value
Input-side inertia	$J_i$	$1.90\times 10^{-4}$ kg m$^{2}$
Spring stiffness	*k*	$118.30\times 10^{3}$ N/m
Lever arm length C	$L_C$	0.056 m
Output link length	$L_D$	0.0645 m
Input viscous fric. coefficient	$c_{v}$	0.262
Input Coulomb fric. coefficient	$c_{c}$	0.145 N
Desired input-side inertia	$J_{i,d}$	$3.80\times 10^{-5}$ kg m$^{2}$
Virtual stiffness	$k_c$	100 N m/rad
Virtual damping	$d_c$	1 N m s/rad

#### Zero-torque mode

**Fig. 6:**
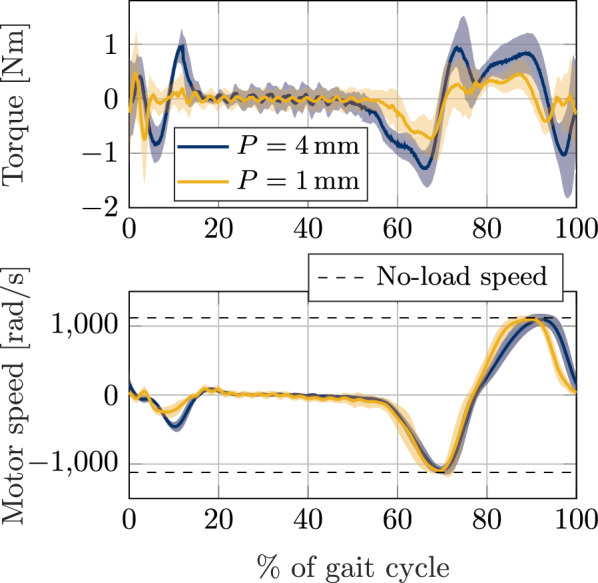
Torque and motor shaft speed measurements throughout 60 gait cycles of a user walking on a treadmill at 0.58m/s wearing the knee exoskeleton in zero-torque mode with two pretension configurations: P=4mm (shown in blue) and P=1mm (shown in yellow). Mean and SD are shown as line and shaded area, respectively. Torque is computed from eq. (13) with measured deflection and known pretension value. Data RMS: 0.30 N m for P = 4 mm and 0.10 N m for P = 1 mm. The lower pretension configuration attains a lower residual torque which might affect user perception. Motor shaft speed measurements showcase the limitation of the motor in terms of acceleration and speed to maintain the desired zero torque during swing

Considering the strategy presented in Sect. "Fault-tolerant torque control", a fixed zero-torque support $\tau _{o,d}=0$ is obtained when $\varphi _{i,d}=\varphi _o$. This should be achievable in dynamic conditions with a fast enough response from the motor. However, in practice, the motor is limited both in maximum speed and acceleration, which might lead to deviations from the desired zero-torque reference. Figure 6 shows the residual torque measurements and motor shaft speed (before transmission) throughout 60 gait cycles of the user walking with the exoskeleton in zero-torque mode with two different pretension values (mean and SD shown as line and shaded area, respectively). It is obvious that the residual torque is higher during swing, where the output position has a faster motion. With a pretension value of $P={4}$ mm, the root mean square (RMS) of the residual torque measurements was 0.30 N m; and for a pretension value of $P={1}$ mm, the obtained RMS was 0.10 N m. A lower pretension value attains a lower residual torque, which is to be expected since the system becomes inherently softer. This, in turn, might affect user perception as a softer configuration might lead to a more transparent interaction in zero-torque control. From the motor shaft speed measurements, it is noticeable that the higher torque deviations occur at points where direction changes and when the motor saturates at its no-load speed. This showcases the limitations of the motor in terms of acceleration and speed. Higher walking speeds could then increase the residual torque during swing, and seriously affect user perception.

#### Support mode

**Fig. 7:**
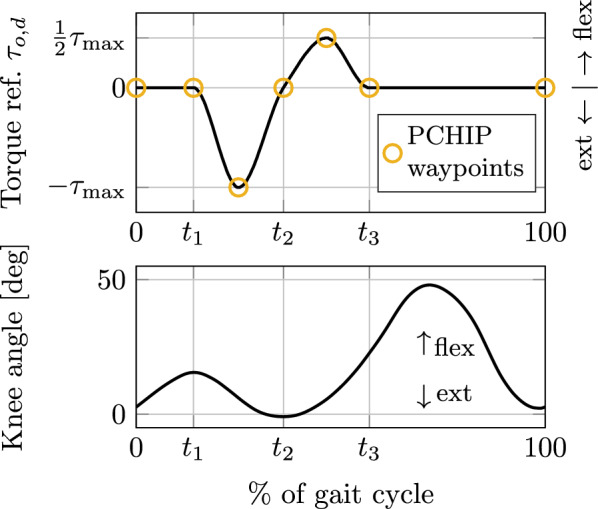
Torque profile for walking support (top) compared to characteristic sagittal plane knee joint angles during a single gait cycle (bottom), reproduced from [39]. Extension support with a peak torque of $\tau _\text {max}$ is provided during early stance and flexion support with a peak torque of $\frac{1}{2}\tau _\text {max}$ is provided during late stance. No support is provided during swing. The profile is characterized by timing parameters $t_1$, $t_2$, and $t_3$. The curve is generated using a Piecewise Cubic Hermite Interpolating Polynomial (PCHIP) with waypoints marked with yellow circles

Biomechanical joint torque data from walking experiments [36, 37] showed that the knee presents a peak of extension torque during early stance, followed by a peak of flexion torque during late stance. Accordingly, the torque profile used in [38] for knee exoskeleton support was implemented in this work. The applied torque profile is shown in Fig. 7. It is characterized by timing parameters $t_1$, $t_2$, and $t_3$, and a support level $\tau _\text {max}$, which corresponds to the peak of extension torque. To reduce the number of parameters, the peak of flexion torque is kept at half of the support level, i.e., $\frac{1}{2}\tau _\text {max}$. The curve is generated using a Piecewise Cubic Hermite Interpolating Polynomial (PCHIP) with waypoints at $(0,0),(t_1,0),(\frac{t_2+t_1}{2},-\tau _\text {max}),(t_2,0),(\frac{t_3+t_2}{2},\frac{1}{2}\tau _\text {max}),(100,0)$. It is important to mention that the provided torque profile does not necessarily correspond to a biomechanically optimal trajectory for an intended purpose, e.g., metabolic cost reduction or rehabilitation, nor was the intention of this work to determine an optimal torque profile. Instead, it serves as a scenario of close interaction to test fault tolerance and user experience.

During experimentation, faults are emulated by lowering the pretension value *P* from its initial value of 4 mm ($P=100\%$) to different fault levels: Low Fault ($P=75\%$), Mid Fault ($P=50\%$), and High Fault ($P=25\%$). Furthermore a *compensated fault* condition was set when the control strategy utilizes the actual value of *P* for calculations, and an uncompensated fault condition was set by utilizing the initial pretension ($P=100\%$) for calculations.

**Fig. 8:**
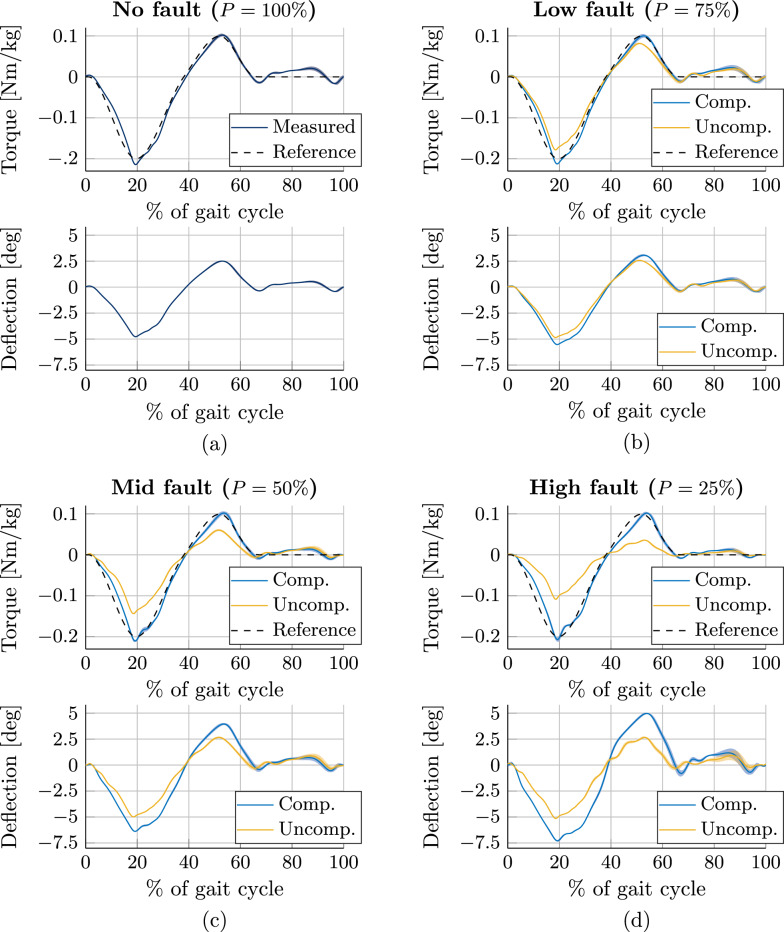
Torque and deflection measurements throughout 60 gait cycles of a user walking on a treadmill at 0.58 m/s wearing the knee exoskeleton providing a torque support profile in four configurations: (**a**) no fault, (**b**) low fault, (**c**) mid fault, and (**d**) high fault. Mean and SD shown as line and shaded area, respectively. Torque is computed from Eq. (13) with measured deflection and known pretension value. Measurements with uncompensated faults (in yellow) show that deflection remains similar throughout all conditions, while the torque level degrades as the fault increases in severity. Measurements with compensated faults (in blue) show that torque levels are maintained close to the reference by increasing deflection, achieving fault tolerance

Figure 8 shows the results of the torque tracking with all different fault conditions throughout 60 gait cycles (mean and SD shown as line and shaded area, respectively). From the plots, it can be seen that for uncompensated faults, the obtained deflection remains similar throughout all fault conditions. Due to the lower stiffness when faults are emulated, the obtained output torque deviates from the reference, with the deviation increasing as the fault becomes more severe. In other words, uncompensated elastic faults lower the torque support level with respect to the fault severity. When faults are compensated, the obtained deflection increases in order to attain the torque reference. That means that higher fault severity requires higher deflection in order to maintain the desired torque level. This might also be a limiting factor, particularly for higher walking speeds, as the saturation of the motor speed might not allow to track the reference torque accurately. Special care should also be taken to avoid damaging the actuator if mechanical limits in position are reached. Yet, it is clear that the torque assistive control strategy is capable of maintaining the torque support level in the presence of faults, effectively achieving fault tolerance.

### User study design

A user study was conducted using the knee exoskeleton on healthy participants during walking on a treadmill. The objective of the study was to investigate how user experience changes in torque levels due to elastic faults during physical interaction with the exoskeleton. The study was conducted with 10 participants (5 women, 5 men) with an average age of ${26.70 \pm 2.06}$ years and an average weight of ${68.20 \pm 16.47}$ kg. Informed consent was obtained from all participants involved in the study. The experimentation was conducted in accordance with the Declaration of Helsinki 2008 and a vote from the Ethics Commission at TU Darmstadt (EK 18/2017). A technician from Sanitätshaus OrthoPoint supervised the orthopedic soundness of the knee exoskeleton and provided suggestions and guidelines for the adjustment of the knee exoskeleton. Accordingly, the leg attachments were individually fitted for each participant.

**Fig. 9:**
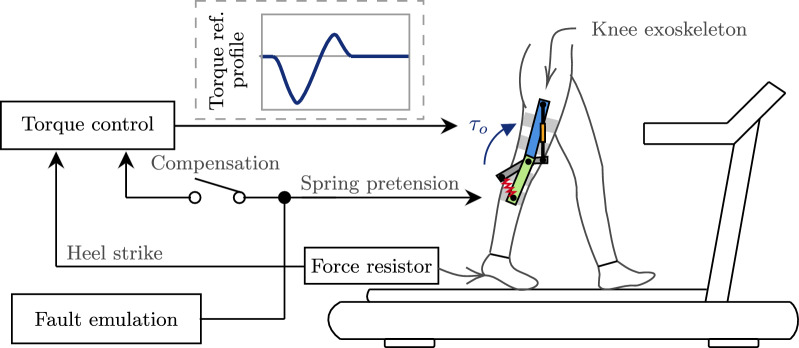
Schematic of experimental setup for the user study on user perception. A controller commands a torque profile based on the user’s gait (synchronized by heel strike). Elastic faults are emulated by altering the spring pretension of the actuator. Faults are compensated when the controller knows the precise altered pretension value, and the torque is corrected

The experimental setup is the same as described in Sect. "Functional evaluation experiments" and represented in Fig. 9. The torque profile shown in Fig. 7 was utilized as torque reference for the control strategy implemented in the knee exoskeleton, while an instrumented insole was used to detect heel strike and synchronize the beginning of each gait cycle. The walking speed was kept fixed at 0.58m/s for all participants to avoid introducing high residual torque during swing, as discussed in Sect. "Zero-torque mode". A repeated-measures experiment was devised with two independent variables (IV). The first IV is the fault severity, which is defined by the value of the pretension *P* with respect to its baseline value: Low Fault ($P=75\%$), Mid Fault ($P=50\%$), and High Fault ($P=25\%$). The second IV is the fault compensation, which is either *compensated* or *uncompensated*. To investigate the effects of both IVs, we implemented a 3x2 repeated-measures design, such that participants experienced 6 different walking conditions in a random order. The dependent variables are the levels of support, stiffness, comfort, and trust perceived by the users at each condition. The configuration where no fault occurs ($P=100\%$) is considered the *baseline walking condition* and serves as a comparison condition for each of the 6 faulty walking conditions.

When the torque profile is applied, participants should be able to distinguish the torque support level depending on the maximum effective torque provided. At the same time, the perception of stiffness, which is expected to be closely related to force perception [30], and walking comfort might also depend on the support level. Furthermore, trust is also an important aspect to measure in the presence of faults, as it allows to understand which aspects contribute to understanding user experience. The hypotheses of the repeated-measures study were:

*Hypothesis 1a: *Participants will perceive changes in support level when faults are uncompensated but not when they are compensated.

*Hypothesis 1b: *Participants will perceive changes in stiffness when faults are uncompensated but not when they are compensated.

*Hypothesis 1c: *Participants will perceive changes in comfort when faults are uncompensated but not when they are compensated.

*Hypothesis 2: *Trust is scored higher for compensated faults than for the uncompensated conditions.

**Fig. 10:**
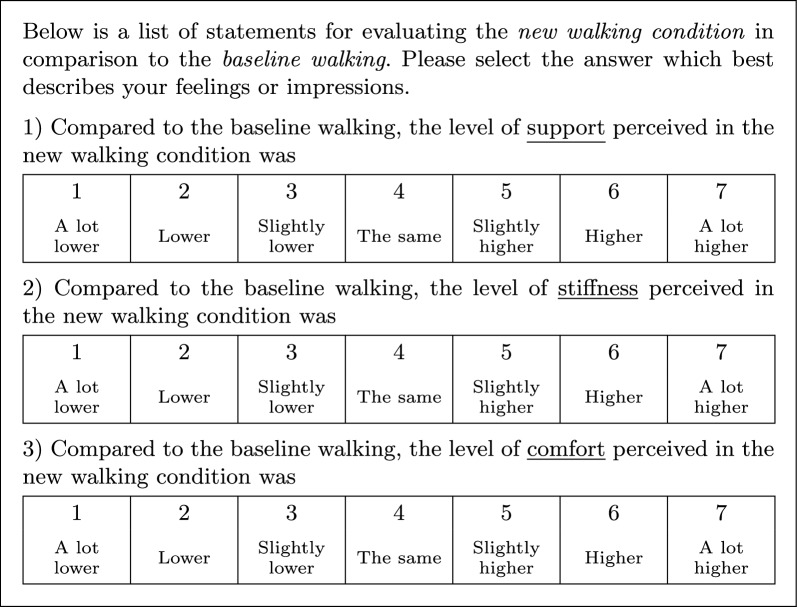
Comparison questionnaire. Compares levels of support, stiffness, and comfort

**Fig. 11:**
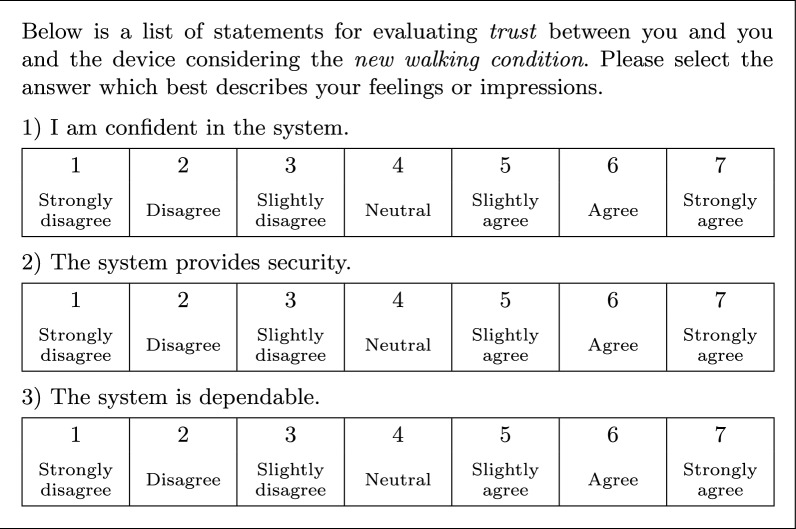
Trust questionnaire. Evaluates confidence, security, and dependability. Extracted from [40]

Hypotheses 1a, 1b, and 1c required that participants compared each condition with the baseline condition, while hypothesis 2 required that participants evaluated their perceived trust after each condition. To achieve this in the repeated-measures study, participants walked with the baseline walking condition for 30 s, followed by 30 s of the faulty condition. To safeguard the mechanical soundness of the pretension altering mechanism, 10 s were left between both walking conditions where the participant stopped walking and the pretension was altered. After each repeated measure, the participant filled out the comparison questionnaire, shown in Fig. 10, which evaluates the perceived level of support, stiffness, and comfort between the baseline and the new condition using a Likert scale of comparison: 1 : *a lot lower*, $2 \!=\!$ *lower*, $3 \!=\!$ *slightly lower*, $4 \!=\!$ *the same*, $5 \!=\!$ *slightly higher*, $6 \!=\!$ *higher*, $7 \!=\!$ *a lot higher*; and the trust questionnaire, shown in Fig. 11, extracted from Jian et al. [40], which evaluates trust in terms of confidence, security, and dependability with a Likert scale of agreement: $1 \!=\!$ *strongly disagree*, $2 \!=\!$ *disagree*, $3 \!=\!$ *slightly disagree*, $4 \!=\!$ *neutral*, $5 \!=\!$ *slightly agree*, $6 \!=\!$ *agree*, $7 \!=\!$ *strongly agree*.

At the beginning of the study, participants received initial instructions and were requested to don the instrumented heel insole and the knee exoskeleton. Adjustments to the leg attachments were made to ensure that the knee exoskeleton was fitted to the individual body geometry. The participant was asked to walk on the treadmill with the zero-torque mode at a slow walking speed of 0.58m/s. A low torque support profile with $\tau _\text {max}={0.05}$ N m/kg (normalized to the user weight) was provided to the participant. Timing parameters $t_1$, $t_2$, and $t_3$ were initially set to $t_1=10\%$, $t_2=40\%$, and $t_3=60\%$ and subsequently minimally adjusted in a range of $\pm 10\%$ based on verbal response from the participant and observed gait pattern measurements, such that the profile did not greatly disturb the natural gait of the participant. This adjustment was not meant to optimize the timing parameters, instead, it was applied to find a good compromise for each participant to conduct the study effectively.

Moreover, a *method of adjustment* experiment was conducted where participants were able to manually adjust the support level by increasing or decreasing $\tau _\text {max}$ using a provided touchpad. Participants were told to reach a support level that was *noticeable but comfortable*. The method of adjustment was performed three times, starting from three different support levels in a random order: 0.10 N m/kg, 0.20 N m/kg, and 0.30 N m/kg. Participants were not able to see the actual torque level, only to increase or decrease the level through buttons in the touchscreen. The average of the three adjustments was used as the *baseline walking condition*. The method of adjustment served two purposes: it gave an insight of the average support level with which users are comfortable at the selected speed of 0.58m/s, while at the same time allowing participants to familiarize with the perception of different support levels such that they can better distinguish them in the subsequent study.

## User study results

The results of the method of adjustment experiments averaged ${0.13 \pm 0.04}$ N m/kg. The selected support levels, which correspond to the peak extension torques, are comparable to the peak extension torque from biological joint data reported by [36] for a similarly low speed (0.5 m/s).

**Fig. 12:**
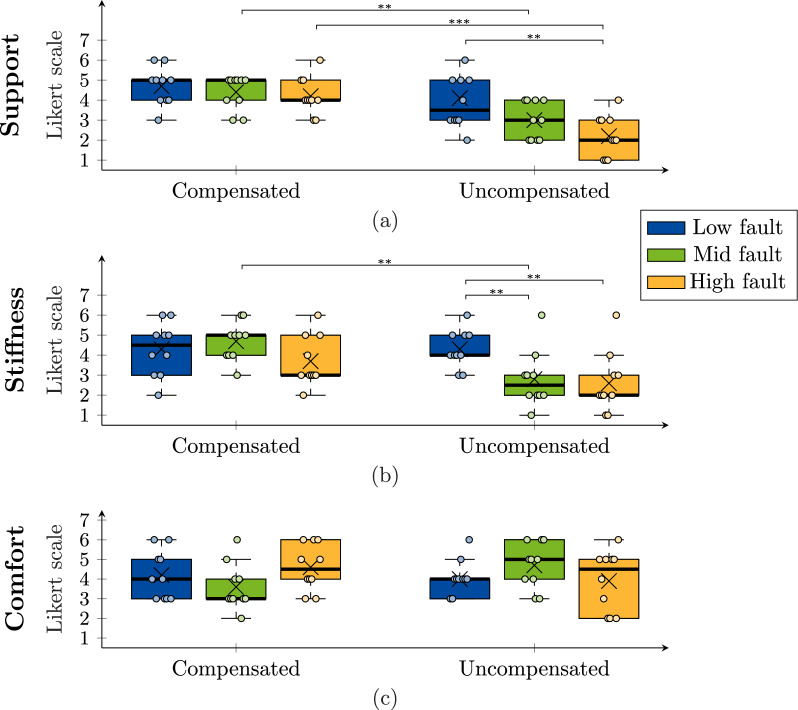
Boxplots of the obtained responses from participants for (**a**) support, (**b**) stiffness, and (**c**) comfort, across combinations of fault severity (low, mid, high) and compensation (compensated or uncompensated), compared to the baseline (no fault). The median is denoted by a thick line, the box edges represent the 25th and 75th percentile, data points are shown as circles, and the average as a cross. Lines above the boxplots indicate significant differences between groups that share an independent variable (Dunn’s test): $$***= p\le 0.01$$, $$**= p\le 0.05$$. Non-significant differences are omitted. We found significant differences in support levels between compensated and uncompensated faults at mid and high severities, as well as between low and high uncompensated fault conditions. This suggests fault compensation helps maintain consistent support levels. Significant differences in stiffness were also found between compensated and uncompensated faults at mid severity, and across all severities in the uncompensated condition, indicating compensation helps maintain expected stiffness levels. Comfort responses showed no significant differences

**Fig. 13:**
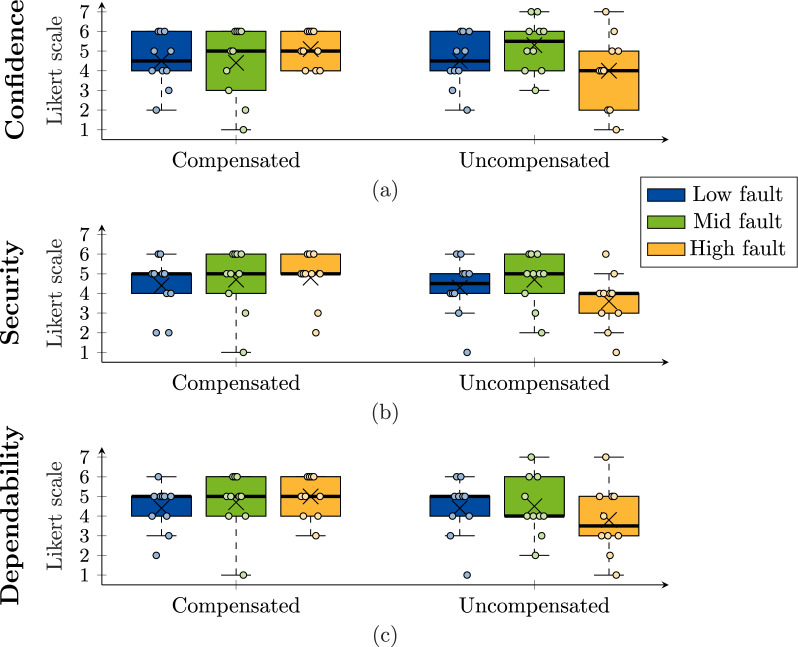
Boxplot of the obtained responses from participants for (**a**) confidence, (**b**) security, and (**c**) dependability, when asked about their agreement after each condition of the repeated-measures study: a combination of fault severity (low, mid, high) and fault compensation (compensated or uncompensated). The median is denoted by a thick line, the box edges represent the 25th and 75th percentile, data points are shown as circles, and the average as a cross. No significant differences were found in the data

The obtained results for the repeated-measures study are summarized in Tables 2 and 3. Figure 12 shows the boxplots of the level comparison for support, stiffness, and comfort; while Fig. 13 shows the boxplots of questions evaluating trust. The normality assumption for all 36 data groups was checked with a Shapiro-Wilk test which showed that 8 out of the 36 groups did not distribute normally ($p<0.046$). For that reason, the differences between the means of the groups for each dependent variable were analyzed with the non-parametric Kruskal-Wallis test [41]. For pairwise comparisons, the Dunn’s test [42] with the Benjamini-Hochberg procedure [43] for the correction of the *p* values was used to determine significant differences between groups that share an independent variable.

**Table 2:** Effect of the IVs on the perception of support, stiffness and comfort when comparing each condition and the baseline

			Fault compensation		*Kruskal-Wallis test*
			Compensated	Uncompens.	
Support	Fault severity	Low	$4.70\pm 0.95$	$3.90\pm 1.29$	$p<0.001$
		Mid	$4.40\pm 0.84$	$3.00\pm 0.94$	
		High	$4.20\pm 0.92$	$2.20\pm 1.03$	
Stiffness	Fault severity	Low	$4.30\pm 1.34$	$4.30\pm 0.95$	$p=0.0022$
		Mid	$4.70\pm 0.95$	$2.80\pm 1.40$	
		High	$3.70\pm 1.25$	$2.60\pm 1.51$	
Comfort	Fault severity	Low	$4.20\pm 1.23$	$4.00\pm 0.94$	$p=0.34$
		Mid	$3.60\pm 1.17$	$4.70\pm 1.16$	
		High	$4.60\pm 1.17$	$3.90\pm 1.52$	

Scale: $1 =$ *a lot lower*, $2 =$ *lower*, $3 =$ *slightly lower*, $4 =$ *the same*, $5 =$ *slightly higher*, $6 =$ *higher*, $7 =$ *a lot higher*. Scores presented as MEAN ± SD

**Table 3:** Effect of the IVs on the perception of trust metrics after each condition

			Fault Compensation		*Kruskal-Wallis test*
			Compensated	Uncompens.	
Confidence	Fault severity	Low	$4.50\pm 1.35$	$4.50\pm 1.35$	$p=0.52$
		Mid	$4.40\pm 1.84$	$5.30\pm 1.34$	
		High	$5.10\pm 0.88$	$4.00\pm 1.89$	
Security	Fault severity	Low	$4.40\pm 1.43$	$4.30\pm 1.49$	$p=0.30$
		Mid	$4.70\pm 1.84$	$4.70\pm 1.34$	
		High	$4.80\pm 1.32$	$3.60\pm 1.43$	
Dependability	Fault severity	Low	$4.40\pm 1.17$	$4.40\pm 1.51$	$p=0.51$
		Mid	$4.70\pm 1.49$	$4.50\pm 1.51$	
		High	$5.00\pm 1.05$	$3.80\pm 1.75$	

Scale: $1 =$ *strongly disagree*, $2 =$ *disagree*, $3 =$ *slightly disagree*, $4 =$ *neutral*, $5 =$ *slightly agree*, $6 =$ *agree*, $7 =$ *strongly agree*. Scores presented as MEAN ± SD

When comparing support, the average results for compensated faults present similar average values across all fault severities. For uncompensated faults, meanwhile, the average results showed a decreasing pattern with respect to an increase in fault severity. The Kruskal-Wallis test showed that there are significant differences between the averages among all 6 conditions ($p<0.001$). The significance levels between groups, obtained with the Dunn’s test and detailed in Fig. 12a, showed that there were no significant differences between fault severities within the compensated conditions, while uncompensated conditions showed significant differences between low and high severities. Moreover, the significance levels between compensated and uncompensated fault conditions show that responses for mid and high severities were significantly different, with uncompensated faults scoring lower.

When comparing stiffness, the Kruskal-Wallis test showed that there are significant differences between the averages among all 6 conditions ($p=0.0022$). The significance levels between groups, obtained with the Dunn’s test and detailed in Fig. 12b, showed that there are no significant differences between fault severities within the compensated conditions, while uncompensated faults showed a decreasing average pattern with respect to fault severity, with significant differences between high-mid and high-low severities. Moreover, there was a significant difference for the mid fault severity when comparing the compensated and uncompensated conditions, with the uncompensated fault scoring lower.

When comparing comfort, there were no discernible patterns in the obtained responses. The Kruskal-Wallis test confirmed this assertion by showing no significant differences among the 6 data groups ($p=0.34$).

When evaluating trust in terms of confidence, security, and dependability, the average results for the uncompensated high fault generally showed lower scores. Yet, the Kruskal-Wallis tests showed no significant differences among the 6 conditions for each dependent variable ($p=0.52$ for confidence, $p=0.30$ for security, and $p=0.51$ for dependability).

## Discussion

The functional evaluation in Sect. "Functional evaluation experiments" showed that fault tolerance can be achieved by the proposed control strategy, by compensating for faults such that a torque support level is maintained throughout several gait cycles despite changes in the spring pretension of the nonlinear elastic actuator that powers a knee exoskeleton.

The user experience study, whose results are presented in Sect. "User study results", explored the impact of elastic faults on user perception when physically interacting with the knee exoskeleton. The results showed significant differences in participant responses when comparing support levels between baseline (no fault) and the different faulty conditions. The comparison of response averages revealed that faulty conditions clearly affected the perception of support levels. While there were no differences in responses for compensated faults, the severity of uncompensated faults led to a decline in perceived support. When comparing responses considering fault compensation, scores were significantly lower for mid and high uncompensated faults. Responses for low severity did not show a significant difference between compensated and uncompensated conditions, suggesting that the difference in torque support caused by the low fault is close to or below the just noticeable difference threshold. Yet, the average and median of responses for the uncompensated fault condition are lower than for the compensated one. These results confirm *Hypothesis 1a*, implying that fault compensation helps maintain the support at comparably similar levels.

The results obtained also presented significant differences when comparing stiffness between baseline (no fault) and the different faulty conditions. However, the impact of fault severity in regards to stiffness, as perceived by the user, is not as distinct as for support. Average responses considering fault severity presented no significant differences for compensated faults, and a decreasing pattern for uncompensated faults. When comparing responses considering fault compensation, scores were significantly lower for the uncompensated mid fault condition. Similar as for support, responses for low severity did not show a significant difference, suggesting that the difference in stiffness caused by the low fault is close to or below the just noticeable difference threshold. Responses for high severity also did not show a significant difference, which suggests that participants were able to distinguish when the system was considerably softer, even when the fault was compensated, likely due to the lower residual torque during swing, as observed in Sect. "Zero-torque mode". This might reflect a limitation of the study, as the residual torque occurring from motor speed saturation seems to influence the perception of stiffness on the participants and confound the results, particularly, for the high fault severity. Yet, the average and median of responses for the uncompensated high fault severity are lower than for the compensated one, which suggests that there was some improvement when faults were compensated, helping to adjust the system to keep stiffness levels closer to what we expected. These results align with *Hypothesis 1b*, and support the argument made by Fu et al. [44] that, in haptic applications, the perception of stiffness is closely related to force perception. The topic of stiffness perception could still be further explored by extending the methods to consider stiffness control profiles [45] or a combination of stiffness and torque segments [38], which could exploit the adaptable virtual stiffness and virtual equilibrium position introduced from impedance control. Furthermore, evaluating the impact of changes in stiffness on the users’ gait, particularly considering the motor limitations described in Sect. "Zero-torque mode", could also be explored in a long exposure user study similar to the one in [46].

Results also showed no significant difference when comparing comfort levels between baseline and faulty conditions. This rejects *Hypothesis 1c* and implies that the perception of comfort is not solely dependent on the torque level. In literature, comfort in exoskeletons can be related to the reduction of muscle fatigue through support [47]. However, this is generally perceived only after an extended use of the device and might be affected by discomfort in the interface between the exoskeleton and the body, or limitations of movement [47]. Moreover, comfort might also be affected by design factors such as the weight of the device [48], or the timing of the support pattern [49].

Finally, results showed no significant difference for the three categories of trust: dependability, security, and confidence. This rejects *Hypothesis 2* and underlines that trust is a complex multi-dimensional concept that is not directly related to the support level in the selected scenario. From a functional perspective, trust is related to the perception of reliability [50]. Faults, as considered in this study, do not severely affect the functioning of the exoskeleton, nor do they hinder the walking performance of an able-bodied person, and thus might not have a severe impact on the perceived reliability. The study of trust in human-robot interaction frequently encounters methodological confounds that undermine the robustness of the findings [51]. In the presented study, the low impact of faults on the task at hand might have contributed to a limited influence on participants’ trust perception. Future research could adopt different pHRI scenarios that provide deeper insights into the interaction between trust and faulty conditions.

## Conclusions

This article proposes a fault-tolerant torque assistive control strategy based on an established impedance control method and presents a user experience study that investigated the user perception of support, stiffness, comfort, and trust during physical human-robot interaction while walking with a knee exoskeleton under different faulty conditions. A functional evaluation demonstrated the feasibility of using analytical methods to compensate for elastic faults, ensuring a desired torque support profile. The user experience study, conducted with 10 participants, showed that fault tolerance can be achieved in practice by compensating for faults and maintaining comparable levels of perceived torque support and stiffness. Meanwhile, results from the user study showed that comfort and trust measures seem not to be directly affected by torque levels.

The findings of this study are important for understanding the role of human perception for the control of wearable robots. They indicate that participants are able to perceive changes in torque levels caused by internal faults, which can be mitigated through fault compensation. Yet, the overall user perception is also influenced by other factors such as ergonomics, timing, and motor performance.

Future work could expand this research by introducing human-in-the-loop optimization methods [29] in combination with user preference and biomechanical data to optimize the torque profile based on a performance metric, and evaluate walking comfort. Moreover, future research could also consider short term faults or walking disturbances to evaluate the ability of the control method to recover operation. Finally, we expect that the methods and findings outlined in this article will serve to improve user satisfaction and dependability of wearable robots.

## Data Availability

The datasets generated during the current study are not publicly available due to privacy restrictions. Access to the software is available from the corresponding author upon request.
